# Impact of air pollution on breast cancer incidence and mortality: a nationwide analysis in South Korea

**DOI:** 10.1038/s41598-020-62200-x

**Published:** 2020-03-25

**Authors:** Jeongeun Hwang, Hyunjin Bae, Seunghyun Choi, Hahn Yi, Beomseok Ko, Namkug Kim

**Affiliations:** 1 Department of Medicine, University of Ulsan College of Medicine, Seoul, Republic of Korea; 2 Department of Convergence Medicine, University of Ulsan College of Medicine, Asan Medical Center, Seoul, Republic of Korea; 3 Asan Institute for Life Sciences, Asan Medical Center, Seoul, Republic of Korea; 4 Department of Breast Surgery, University of Ulsan College of Medicine, Asan Medical Center, Seoul, Republic of Korea; 5 Department of Radiology and Research Institute of Radiology, University of Ulsan College of Medicine, Asan Medical Center, Seoul, Republic of Korea

**Keywords:** Breast cancer, Epidemiology, Risk factors

## Abstract

Breast cancer is one of the major female health problems worldwide. Although there is growing evidence indicating that air pollution increases the risk of breast cancer, there is still inconsistency among previous studies. Unlike the previous studies those had case-control or cohort study designs, we performed a nationwide, whole-population census study. In all 252 administrative districts in South Korea, the associations between ambient NO_2_ and particulate matter 10 (PM_10_) concentration, and age-adjusted breast cancer mortality rate in females (from 2005 to 2016, N_mortality_ = 23,565), and incidence rate (from 2004 to 2013, N_incidence_ = 133,373) were investigated via multivariable beta regression. Population density, altitude, rate of higher education, smoking rate, obesity rate, parity, unemployment rate, breastfeeding rate, oral contraceptive usage rate, and Gross Regional Domestic Product per capita were considered as potential confounders. Ambient air pollutant concentrations were positively and significantly associated with the breast cancer incidence rate: per 100 ppb CO increase, Odds Ratio OR = 1.08 (95% Confidence Interval CI = 1.06–1.10), per 10 ppb NO_2_, OR = 1.14 (95% CI = 1.12–1.16), per 1 ppb SO_2_, OR = 1.04 (95% CI = 1.02–1.05), per 10 µg/m^3^ PM_10_, OR = 1.13 (95% CI = 1.09–1.17). However, no significant association between the air pollutants and the breast cancer mortality rate was observed except for PM_10_: per 10 µg/m^3^ PM_10_, OR = 1.05 (95% CI = 1.01–1.09).

## Introduction

Breast cancer is the most frequently diagnosed cancer among women worldwide^1^, and is rapidly increasing in industrialized countries and urban areas. In South Korea, breast cancer is the second most common after thyroid cancer and has annually increased by 6.1% from 1999 to 2014^2,3^. Increased exposure to environmental female hormones is considered to affect the rise of breast cancer incidence. In addition, hormone-dependent cancer is increasing in industrialized countries^1,4^.

There is growing evidence indicating that air pollution is a risk factor for breast cancer. Nitrogen oxides (NO_2_ and NO_x_)^5–9^, fine particulate matters (PM_10_ and PM_2.5_)^10,11^, and polycyclic aromatic hydrocarbons (PAHs)^12,13^ are reported to associate with breast cancer incidence. The physiological mechanisms by which air pollutants affect breast cancer are largely explained in two ways. First, air pollutants may directly cause genetic mutations, as they are carcinogenic^14,15^. Second, air pollutants may affect breast cancer incidence by increasing breast density, which is known to be a risk factor. Yaghjyan *et al*. reported an association between exposure to PM_2.5_, O_3_, and mammographic breast density^16^. Female hormones affect breast density, and some air pollutants are known to exhibit endocrine-disrupting properties, including xenoestrogens^17^. However, in the Danish Diet, Cancer and Health cohort (1993–1997) study, little evidence of association between traffic-related air pollution exposure and breast density was found^18^. Hung *et al*. reported a positive association between high levels of PM_2.5_ and breast cancer mortality rate, by considering PM_2.5_ as a marker for polycyclic aromatic hydrocarbons^19^.

There is still an inconsistency among the evidence for the association between air pollution and breast cancer risk^20^. Though Crouse *et al*.^6^ found a positive association between NO_2_ concentration and the breast cancer incidence by 95% confidence, subsequent studies found positive associations with partial^8,10,11^, marginal or null^8,10,11,21–23^ statistical confidence. For example, Hystad *et al*. found a significant association between NO_2_ concentration and premenopausal breast cancer while marginal association were found between NO_2_ and postmenopausal breast cancer in Canadian women^8^. Conversely, Anderson *et al*. found significant positive associations between the incidence of postmenopausal breast cancer in European women and NO_x_, and nickel in PM_10_, but found only suggestive evidence with PM_2.5_, PM_10_, PM_coarse_, NO_2_, and nickel in PM_2.5_, vanadium in PM_2.5_, and vanadium in PM_10_^10^. Incorporating more cases or expanding the cohort population may clarify the evidence, though such efforts would demand resources. There also were ecological studies on the association between breast cancer and air pollution^5,7,9^. Chen *et al*.^5^ and Wei *et al*.^9^ aggregated air pollutant emission data and age-adjusted breast cancer incidence rates in Surveillance, Epidemiology, and End Results Program of the United States National Cancer Institute that covered 199 counties and approximately 100,000 or less female population and found positive correlations. Datzman *et al*.^7^ found significant positive associations between NO_2_, PM_10_, and breast cancer incidence by using healthcare data from a local insurance corporation in Germany that covers approximately 1,000,000 female population and 9,577 incidences.

We performed an ecological study to investigate the associations between CO, NO_2_, SO_2_, O_3_, PM_10_, and breast cancer incidence and mortality rate, in the whole of the 252 administrative districts in South Korea. South Korea has a unique national healthcare insurance system with the full coverage of its 52 million citizens and provides mammography-based breast cancer screening service for all female citizens with age ≥40 in every two-years^24^. The lifetime screening rate for breast cancer in females with age ≥40 was as high as 83.1% by 2013^25^. Among the Organization for Economic Co-operation and Development nations in 2012, South Korea have the eighth highest age-adjusted breast cancer incidence rate of 52.1 per 100,000, but have the lowest mortality rate of 6.1 per 100,000^26^. The full coverage of national healthcare insurance and the high screening rate may have facilitated appropriate medical intervention for breast cancer. The relative 5-year survival rate was higher than those of the United States and Japan in similar years: 92.3% in South Korea 2011–2015, 91.1% in the United States 2007–2013, 91.1% in Japan 2006–2008, respectively^26^. Moreover, the national breast cancer screening service followed up all breast cancer patients in South Korea by residential address and exhaustively recorded the incidence and mortality rate^24^. These unique healthcare settings surrounding breast cancer make South Korea a natural testing ground to investigate associations between air pollution and breast cancer incidence and mortality rate.

## Results

**Figure 1:**
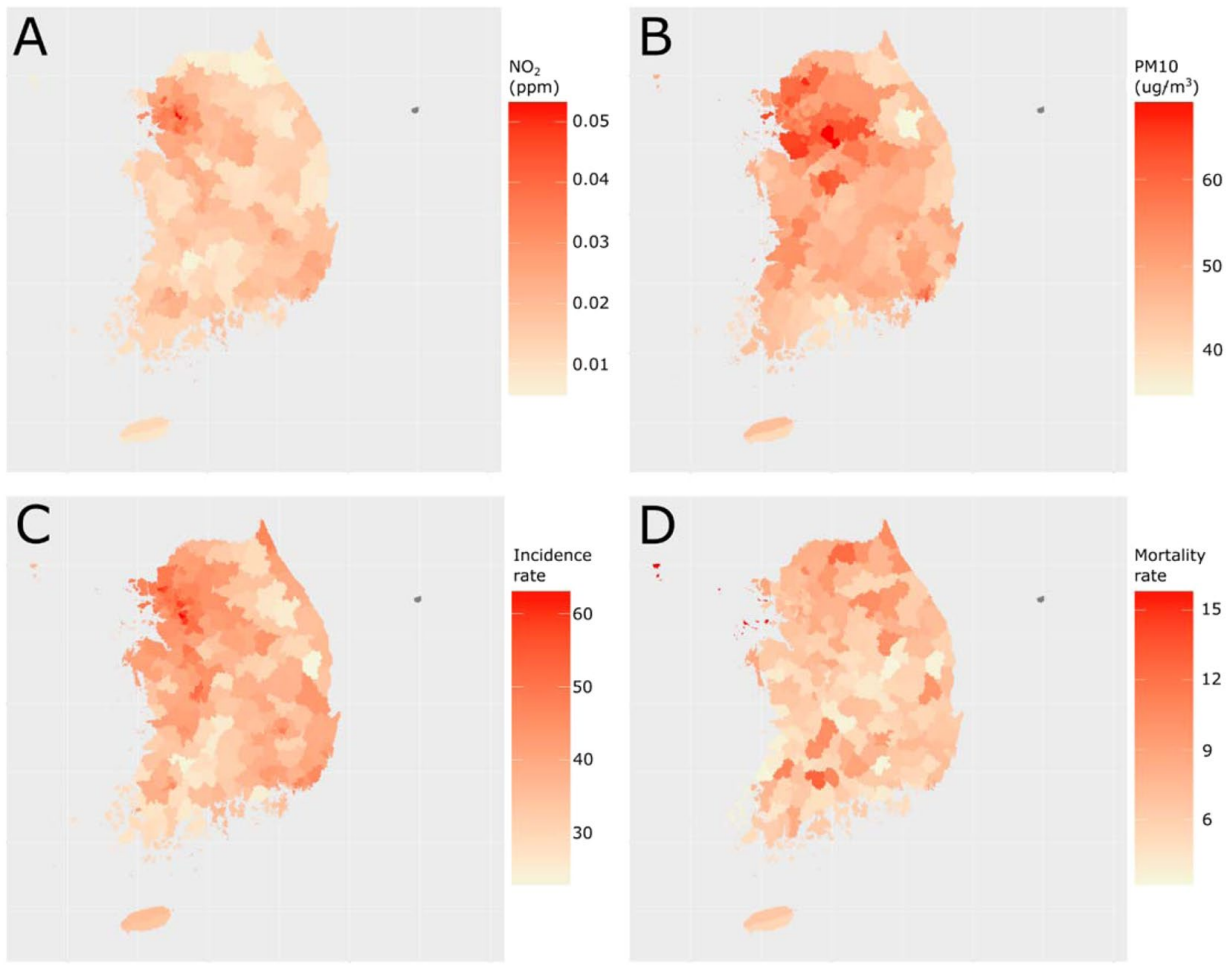
Concentrations of NO_2_ and PM_10_, and age-adjusted breast cancer incidence and mortality rates portrayed on the South Korean map. (**A**) NO_2_ concentration in average of the study period (2004–2016), (**B**) PM_10_ concentration in average of 2004–2016, (**C**) age-adjusted breast cancer incidence rate in average of 2004–2013, and (**D**) age-adjusted breast cancer mortality rate in average of 2005–2016, in South Korea.

Characteristics of the observed districts throughout the study period are shown in Table 1. In Fig. 1, NO_2_ and PM_10_, concentrations and age-adjusted breast cancer incidence and mortality rates are portrayed on the South Korean map. Korean female population as of 2010 (in the middle of the study period) was 24,149,865. The total number of breast cancer incidence in 2004–2013 was 133,373, and the total number of deaths by breast cancer in 2005–2016 was 23,565.

**Table 1:** Characteristics of the study area.

Characteristic	Numbers or median (1^st^–3^rd^ quartile range)
Number of districts	251
Age-adjusted breast cancer mortality rate (per 100,000)^a^	6.60 (5.65–7.50)
Age-adjusted breast cancer incidence rate (per 100,000)^b^	41.6 (35.2–46.5)
Carbon monoxide (ppb)^c^	531 (462–597)
Nitrogen dioxide (ppb)	19.9 (14.1–27.6)
Sulfate dioxide (ppb)	4.93 (4.04–5.65)
Ozone (ppb)	25.0 (22.0–28.6)
PM_10_ (µg/m^3^)	49.2 (45.6–54.2)
Altitude (m)	124 (58.9–220)
Population density (per km^2^)	410 (110–6394)
Higher-education rate^d^ (%)	29.1 (16.8–36.9)
Smoking rate^e^ (%)	25.1 (23.4–26.8)
Obesity rate^f^ (%)	22.4 (20.9–24.4)
Parity^g^	2.5 (2.2–2.7)
Unemployment rate^h^ (%)	2.7 (2.2–3.8)
Breast feeding rate^i^ (%)	0.883 (0.862–0.898)
Oral contraceptive usage rate^j^ (%)	0.131 (0.106–0.141)
GRDP per capita^k^ (million won)	22.8 (16.7–28.8)

From 2005 to 2016, the female population in all 251 South Korean administrative districts, Si-Gun-Gus, were studied. Korean female population, as of 2010 (in the middle of the study period) was 24,149,865.

^a^Total number of deaths by breast cancer from 2005–2016 was 23,565. The annual raw mortality rates throughout the study period were adjusted for each district’s age distribution to the standard Koran female population in 2010.

^b^Breast cancer incidence data were surveyed by the Korean government in every 5 years. The number of breast cancer incidence in the nationwide female population in 2004–2008 period was 54,859, and in 2009–2013 period was 77,952.

^c^Air pollution data in 2004–2016 were accessed via AirKorea database in daily mean concentrations according to the positions of monitoring stations. An interpolation model based on a geographical information system was applied to yield average air pollutant concentration throughout the study period of the corresponding districts.

^d^Rate of >15-year-old women with equal to or higher than a college education in 2010.

^e^Rate of current female smokers adjusted by the age of the national standard female population in 2010.

^f^Rate of females with BMI > 25 adjusted by the age of the national standard female population in 2010.

^g^Number of childbirth per married >15-years-old women in 2010.

^h^Rate of unemployed >15-years-old women in 2010.

^i^Rate of females with breastfeeding history in 2010.

^j^Rate of females with oral contraceptive usage in 2010.

^k^Gross Regional Domestic Product per capita in 2011.

In multicollinearity analysis among the air pollutants, the NO_2_ and O_3_ concentrations were colinear with a correlation coefficient of −0.862. O_3_ concentrations were excluded from the multivariable model due to its lesser correlation (0.659) with the breast cancer incidence rate than that of NO_2_ concentrations (0.774). Both NO_2_ and O_3_ concentrations poorly associated with the breast cancer mortality rates (<0.2 for both). Other air pollutants, CO, SO_2_, and PM_10_ showed little evidence of collinearity with <0.7 correlation coefficients. Among the confounding factors, higher education rate was collinear with population density, parity, unemployment rate, breastfeeding rate, and oral contraceptive usage rate with >0.7 correlation coefficients. Among those, only higher education rate was included in the model due to its strongest association with the breast cancer incidence rate (0.832). Altitude, smoking rate, obesity rate, gross regional domestic product (GRDP) per capita, and higher education rate were not significantly collinear among each other, so those five confounding factors were incorporated to the beta regression model in estimating the odds ratio (OR) of CO, NO_2_, SO_2_, PM_10_ concentrations for breast cancer incidence and mortality rates as covariates.

Table 2 shows the OR estimates by single-pollutant and multi-pollutant multivariable linear regression models adjusted for altitude, higher-education rate, smoking rate, obesity rate, and GRDP per capita. In single-pollutant models, all of the four pollutants, CO, NO_2_, SO_2_, and PM_10_ were significantly associated with breast cancer incidence. For example, a district with 10 ppb higher NO_2_ concentration suffered from higher OR of breast cancer incidence by 1.14 (95% Confidence Interval: 1.12–1.16). In multi-pollutant models, all of the four pollutants remained associated with the breast cancer incidence rate when additionally adjusted with the other three pollutants. On the other hand, air pollutants’ concentrations and the breast cancer mortality rates exhibited subtler associations. CO, NO_2_, and SO_2_ showed positive but not significant associations, both in single- and multi-pollutant models. Only PM10 exhibited significant associations with the breast cancer mortality rate both in single- and multi-pollutant models, but with smaller ORs than those with the breast cancer incidence rate.

**Table 2:** Odds Ratios (OR) from multivariable beta regression models with air pollutants controlled for altitude, higher-education rate, smoking rate, obesity rate, and GRDP per capita.

	Breast cancer incidence rate OR (95%CI)	Breast cancer mortality rate OR (95%CI)
**Single-pollutant**		
CO (per 100 ppb)	**1.08 (1.06–1.10)**	1.02 (0.987–1.05)
NO_2_ (per 10 ppb)	**1.14 (1.12–1.16)**	1.02 (0.983–1.05)
SO_2_ (per 1 ppb)	**1.04 (1.02–1.05)**	1.02 (0.991–1.04)
PM_10_ (per 10 µg/m^3^)	**1.13 (1.09–1.17)**	**1.05 (1.01–1.09)**
**Multi-pollutant**		
CO with NO_2_, SO_2_, and PM_10_	**1.09 (1.07–1.12)**	1.02 (0.989–1.05)
NO_2_ with CO, SO_2_, and PM_10_	**1.14 (1.11–1.16)**	1.03 (0.985–1.06)
SO_2_ with CO, NO_2_, and PM_10_	**1.04 (1.02–1.06)**	1.01 (0.989–1.03)
PM_10_ with CO, NO_2_, and SO_2_	**1.13 (1.08–1.17)**	**1.04 (1.00–1.09)**

## Discussion

NO_2_ and PM_10_ concentrations were significantly and positively associated with the breast cancer incidence rate in South Korean female population. This result is consistent with previous studies that reported significantly higher risk of breast cancer incidence^5,7,9,11^, and is partly consistent with studies that reported suggestive^6,8,10,12,14,15,27^ or null^11,21–23,28^ associations between air pollutants and breast cancer. Our study adds evidence of a significant positive association to the aforementioned studies. It is also important to note that our finding is based on region-based national census data that encompassed the entire female population of a country (24,149,865 in 2010) for more than 10 years, including the complete set of diagnosed breast cancer incidences (132,811) and deaths (23,565) during that period. This region-based national census data could covary out possible confounders, including the data-collection method and any other unknown factors. This adds a new layer of evidence for the association between air pollution and breast cancer incidence rates to the previous studies that had case-control or cohort settings. Differences in the significance of the positive association in our study and aforementioned studies may be due to the relatively severer air pollution in South Korea (Table 1) compared with the air pollution measured in European^10,23^ or North American cohorts^6,8,11,14,22,27^. If so, countries with severe air pollution, such as China or India, may also exhibit significant positive associations that are similar to our result. Differences in ethnic composition, diet, and culture may have also played a role. In addition, subsequent studies will be needed to uncover the underlying physiological mechanisms and pathways in these associations. As previous studies^14–16,19^ suggest, NO_2_ and PM_10_ may exert both endocrine-disrupting and carcinogenic properties. Regions with severe air pollution may co-localize with other endocrine-disrupting agents or carcinogens, thereby exhibiting a harmful association with breast cancer incidence.

On the other hand, no significant association was found between air pollution and breast cancer mortality rates except PM_10_. The breast cancer incidence and mortality rates are positively, but weakly associated in South Korea, with a Pearson’s correlation coefficient of 0.150 (p-value: 0.0173). This weak association implies that there are some districts with higher mortality rates than expected with their incidence rates. Many of these districts are located in rural areas, supporting an idea that they are underserved by the healthcare system (including late-detection or late-diagnosis) but we did not find significant differences between these low-incidence-high-mortality districts and the other districts. After the breast cancer diagnosis, a patient may have various treatment and management options that considerably affect the mortality rate, and those are hard to parameterize in a census-based study setting. For instance, living in a polluted urban area may lead to a high incidence rate but not to a high mortality rate, by providing better access to healthcare resources than other parts of the country. Similarly, the higher-education rate is positively correlated with the incidence rate, but not with mortality rate. Higher education is usually associated with a Westernized diet pattern, fewer childbirths, prolonged time-to-first pregnancy, and less breastfeeding. These factors contribute to a higher risk of breast cancer incidence. On the other hand, higher education may lower the mortality rate by promoting patients to seek better means to fight the disease. There are studies reporting that different education groups have disparate treatment and mortality patterns in South Korea^29^ and China^30^.

Our breast cancer incidence and mortality statistics are based on a validated national census database, encompassing the whole female population in South Korea. Although this makes our study robust, a limitation also arises. Because the unit of analysis was a district, not an individual, some information considered to be important in breast cancer incidence could not be obtained, such as: histologic subtypes, stage at diagnosis, menopausal status, molecular subtypes, mammographic breast density, occupational history, and patient-specific exposure to air pollution. Further research is needed to unveil the interplay of breast cancer, air pollution, and these not-yet-studied risk factors. We did not perform a lag analysis due to the lack of temporal resolution in the breast cancer incidence that had been surveyed every 5 years (in 2004–2008 period and 2009–2013 period, only twice in the study period). Because there was no predefined ‘duration of exposure’ nor a ‘lag-year’ value from an exposure to air pollution to lead to a breast cancer incidence or mortality case, the average of daily air pollutant concentrations throughout the study period in each district was considered to represent the level of air pollution. More frequent survey on the breast cancer incidence would enable time series analysis and may lead to richer implications to the field. Another limitation is the lack of migration history data. We contend that migrations would not significantly flaw the current study because only approximately 10% of the population moved between different districts in South Korea between 2003 and 2013^31,32^, and the average duration of living in the same district, according to the 2005 Census, was about 7.7 years^31,32^.

## Conclusions

Our study suggests a positive association between air pollution and breast cancer incidence, but less definitively with the mortality rate. This region-based, nationwide, whole-population study adds a new layer of evidence for the association.

## Methods

### Ethical approval

Ethical approval was not required because this study was performed using a publicly accessible, national epidemiology database.

### Breast cancer incidence and mortality statistics

Korean Statistical Information Service (KOSIS)^32^ is a publicly accessible database that was used to extract the breast cancer incidence and mortality rate, which was classified by the Korean Standard Classification of Disease codes for “breast cancer”, C50, and corresponding with the same disease category in the 10th revision of International Statistical Classification of Diseases (ICD-10) and Related Health Problems codes. Mortality statistics were provided from 2005–2016, and incidence statistics were from 2004–2013 by the KOSIS database. There are currently 252 Si-Gun-Gus in South Korea. *Si-Gun-Gu* is a level in the Korean administrative-area system, similar to the *county* in the United States. All 252 districts were included in this study, except Ulleung-Gun—a group of islands 130 km away from the east coast, populated by fewer than 10,000 people. The incidence and mortality rates were age-adjusted per 100,000 by the standard populations as of July 1^st^, 2010 in South Korea. Breast cancer incidence or deaths in the male population were too scarce to be included in the study, so only the female population was analyzed.

### Air pollution

Air pollution data throughout the study period and places were acquired from a publicly accessible database. CO, SO_2_, NO_2_, O_3_, PM_10_, and PM_2.5_ concentrations are measured by National Ambient Air Quality Monitoring Information System (NAMIS), and publicly accessible via the AirKorea website. In total, there are 332 measurement stations nationwide. PM_2.5_ was not assessed in the current study because of shortage in measurement stations in the early study period. The average of each pollutant, per day, for each station was collected.

Although the air pollution data are based on the measurement stations, the population, incidence, and death statistics are based on the Si-Gun-Gu district system, which is not directly matched to each other. To match and integrate the datasets, we obtained the latitudes and longitudes of each air pollution measurement station and administrative authorities office as a representative location for each district. Then we estimated the average air pollutant concentrations throughout the study period for each administrative office by linearly interpolating air pollutant measurements from the surrounding three stations. Python programming language version 2.7 (Python Software Foundation, Beaverton, Oregon, United States) was used in the procedure.

Considering the nature of cancer incidence that depends on long-term, cumulative exposure to putative carcinogens, a clear-cut exposure timing may not be determined. Rather, we summarized the mean ambient pollutant concentrations throughout the whole study period (2004–2016).

### Confounding factors

Altitude, population density, higher-education rate, smoking rate, obesity rate, parity, unemployment rate, breastfeeding rate, oral contraceptive usage rate as of the 2010 Census, and gross regional domestic product (GRDP) per capita as of 2011 were accessed for every districts via the KOSIS database and considered as potential confounding factors. The rates are defined as follows: higher education rate (rate of >15-year-old women with equal to or higher education than college education in the district), smoking rate (rate of current female smokers adjusted by the age of the national standard female population), obesity rate (rate of females with BMI >25 adjusted by the age of the national standard female population), parity (number of childbirth per married >15-years-old women), unemployment rate (rate of unemployed >15-years-old women), breastfeeding rate (rate of females with breastfeeding history), oral contraceptive usage rate (rate of females with oral contraceptive usage). Parity, unemployment rate, breastfeeding rate, and oral contraceptive usage rate were provided only per 17 provinces, that is coarser than other covariates provided per 252 districts. For parity, unemployment rate, breastfeeding rate, and oral contraceptive usage rate, districts in the same province were attributed with the same estimates.

### Statistical analysis

Data are shown as median and interquartile range and the 95% confidence interval (95%CI) where applicable. Multivariable beta regression^33,34^ models for the breast cancer incidence rates and mortality rates were built, and odds ratio (OR) of each air pollutant to the incidence and mortality rates were estimated, adjusting for the confounding factors. To estimate the 95% confidence intervals for ORs, a basic bootstrap method was applied. To minimize the multicollinearity in the model, variable pairs with Pearson’s correlation coefficients higher than 0.7 were identified, and variables of lower correlation with the breast cancer incidence rate and mortality rates were excluded from the model. R statistics software version 3.6.2 (R Foundation for Statistical Computing, Vienna, Austria) was used in this study.

## Data Availability

The data that support the findings of this study are available from public databases: Korean Statistical Information Service, http://kosis.kr/; AirKorea, an air quality information system provided by the Korean Ministry of Environment and the Korean Environment Corporation, http://www.airkorea.or.kr/index; SHAPE file of South Korean map available at National Geographic Information Institute of Korea, http://ngii.go.kr.

## References

[1] Torre LA, Islami F, Siegel RL, Ward EM, Jemal A (2017). Global Cancer in Women: Burden and Trend. Cancer Epidem Biomar.

[2] Jung KW (2017). Cancer Statistics in Korea: Incidence, Modality, Survival, and Prevalence in 2014. Cancer Res Treat.

[3] Park EH (2017). Basic Facts of Breast Cancer in Korea in 2014:The 10-Year Overall Survival Progress. J Breast Cancer.

[4] Lopez-Abente, G. *et al*. Time trends in municipal distribution patterns of cancer mortality in Spain. *Bmc Cancer* **14**, 10.1186/1471-2407-14-535 (2014). 10.1186/1471-2407-14-535 PMC4124154 25060700

[5] Chen F, Bina WF (2012). Correlation of white female breast cancer incidence trends with nitrogen dioxide emission levels and motor vehicle density patterns. Breast Cancer Res Tr.

[6] Crouse DL, Goldberg MS, Ross NA, Chen H, Labreche F (2010). Postmenopausal Breast Cancer is Associated with Exposure to Traffic-Related Air Pollution in Montreal, Canada: A Case-Control Study. Environ Health Persp.

[7] Datzmann, T. *et al*. Outdoor air pollution, green space, and cancer incidence in Saxony: a semi-individual cohort study. *Bmc Public Health* **18**, 10.1186/s12889-018-5615-2 (2018). 10.1186/s12889-018-5615-2 PMC5994126 29884153

[8] Hystad P (2015). Exposure to traffic-related air pollution and the risk of developing breast cancer among women in eight Canadian provinces: A case-control study. Environ Int.

[9] Wei YD, Davis J, Bina WF (2012). Ambient air pollution is associated with the increased incidence of breast cancer in US. Int J Environ Heal R.

[10] Andersen Zorana J., Stafoggia Massimo, Weinmayr Gudrun, Pedersen Marie, Galassi Claudia, Jørgensen Jeanette T., Oudin Anna, Forsberg Bertil, Olsson David, Oftedal Bente, Marit Aasvang Gunn, Aamodt Geir, Pyko Andrei, Pershagen Göran, Korek Michal, De Faire Ulf, Pedersen Nancy L., Östenson Claes-Göran, Fratiglioni Laura, Eriksen Kirsten T., Tjønneland Anne, Peeters Petra H., Bueno-de-Mesquita Bas, Plusquin Michelle, Key Timothy J., Jaensch Andrea, Nagel Gabriele, Lang Alois, Wang Meng, Tsai Ming-Yi, Fournier Agnes, Boutron-Ruault Marie-Christine, Baglietto Laura, Grioni Sara, Marcon Alessandro, Krogh Vittorio, Ricceri Fulvio, Sacerdote Carlotta, Migliore Enrica, Tamayo-Uria Ibon, Amiano Pilar, Dorronsoro Miren, Vermeulen Roel, Sokhi Ranjeet, Keuken Menno, de Hoogh Kees, Beelen Rob, Vineis Paolo, Cesaroni Giulia, Brunekreef Bert, Hoek Gerard, Raaschou-Nielsen Ole (2017). Long-Term Exposure to Ambient Air Pollution and Incidence of Postmenopausal Breast Cancer in 15 European Cohorts within the ESCAPE Project. Environmental Health Perspectives.

[11] Reding KW (2015). Breast Cancer Risk in Relation to Ambient Air Pollution Exposure at Residences in the Sister Study Cohort. Cancer Epidem Biomar.

[12] Bonner MR (2005). Breast cancer risk and exposure in early life to polycyclic aromatic hydrocarbons using total suspended particulates as a proxy measure. Cancer Epidem Biomar.

[13] Mordukhovich I (2010). Associations between Polycyclic Aromatic Hydrocarbon-Related Exposures and p53 Mutations in Breast Tumors. Environ Health Persp.

[14] Callahan CL (2018). Lifetime exposure to ambient air pollution and methylation of tumor suppressor genes in breast tumors. Environ Res.

[15] Rodgers KM, Udesky JO, Rudel RA, Brody JG (2018). Environmental chemicals and breast cancer: An updated review of epidemiological literature informed by biological mechanisms. Environ Res.

[16] Yaghjyan, L. *et al*. Association between air pollution and mammographic breast density in the Breast Cancer Surveilance Consortium. *Breast Cancer Res* **19**, 10.1186/s13058-017-0828-3 (2017). 10.1186/s13058-017-0828-3 PMC5382391 28381271

[17] Brody JG, Rudel RA (2003). Environmental pollutants and breast cancer. Environ Health Persp.

[18] Huynh, S. *et al*. Long-term exposure to air pollution and mammographic density in the Danish Diet, Cancer and Health cohort. *Environ Health-Glob* **14**, 10.1186/s12940-015-0017-8 (2015). 10.1186/s12940-015-0017-8 PMC4392475 25879829

[19] Hung LJ (2012). Traffic Air Pollution and Risk of Death from Breast Cancer in Taiwan: Fine Particulate Matter (PM2.5) as a Proxy Marker. Aerosol Air Qual Res.

[20] White AJ, Bradshaw PT, Hamra GB (2018). Air pollution and Breast Cancer: A Review. Curr Epidemiol Rep.

[21] Goldberg MS (2017). The association between the incidence of postmenopausal breast cancer and concentrations at street-level of nitrogen dioxide and ultrafine particles. Environ Res.

[22] Hart JE (2016). Long-term Particulate Matter Exposures during Adulthood and Risk of Breast Cancer Incidence in the Nurses’ Health Study II Prospective Cohort. Cancer Epidem Biomar.

[23] Raaschou-Nielsen, O. *et al*. Air pollution from traffic and cancer incidence: a Danish cohort study. *Environ Health-Glob* **10**, 10.1186/1476-069x-10-67 (2011). 10.1186/1476-069X-10-67 PMC3157417 21771295

[24] Center, N. C. *National Cancer Screening Services*, http://www.ncc.re.kr/.

[25] Suh M (2016). Trends in Cancer Screening Rates among Korean Men and Women: Results of the Korean National Cancer Screening Survey, 2004–2013. Cancer Res Treat.

[26] *GLOBOCAN**2012*, *Estimated cancer incidence, mortality and prevalence worldwide in 2012*, http://globocan.iarc.fr/Pages/fact_sheets_cancer (2012).

[27] Shekarrizfard M (2015). Investigating the role of transportation models in epidemiologic studies of traffic related air pollution and health effects. Environ Res.

[28] Hansen AB (2016). Long-term exposure to fine particulate matter and incidence of diabetes in the Danish Nurse Cohort. Environ Int.

[29] Bahk J, Jang SM, Jung-Choi K (2017). Increased breast cancer mortality only in the lower education group: age-period-cohort effect in breast cancer mortality by educational level in South Korea, 1983–2012. Int J Equity Health.

[30] Liu Y (2017). Influence of occupation and education level on breast cancer stage at diagnosis, and treatment options in China: A nationwide, multicenter 10-year epidemiological study. Medicine (Baltimore).

[31] Kim Ok-Jin, Kim Sun-Young, Kim Ho (2017). Association between Long-Term Exposure to Particulate Matter Air Pollution and Mortality in a South Korean National Cohort: Comparison across Different Exposure Assessment Approaches. International Journal of Environmental Research and Public Health.

[32] Korea, S. *Statistics Korea News*, http://kostat.go.kr/portal/eng/news/3/index.board (2019).

[33] Ferrari SLP, Cribari-Neto F (2004). Beta regression for modelling rates and proportions. J Appl Stat.

[34] Cribari-Neto F, Zeileis A (2010). Beta Regression in R. J Stat Softw.

